# Evaluation of Manganese Chloride’s Effect on Biosynthetic Properties of In Vitro Cultures of *Eschscholzia californica* Cham.

**DOI:** 10.3390/molecules23040971

**Published:** 2018-04-21

**Authors:** Andrea Balažová, Júlia Urdová, František Bilka, Ivana Holková, Branislav Horváth, Vladimír Forman, Pavel Mučaji

**Affiliations:** 1 Department of Cell and Molecular Biology of Drugs, Faculty of Pharmacy, Comenius University, Kalinčiakova 8, 83232 Bratislava, Slovakia; bilka@fpharm.uniba.sk (F.B.); holkova@fpharm.uniba.sk (I.H.); 2 Department of Pharmacognosy and Botany, Faculty of Pharmacy, Comenius University, Odbojárov 10, 83232 Bratislava, Slovakia; urdova5@uniba.sk (J.U.); forman@fpharm.uniba.sk (V.F.); 3 Central NMR Laboratory, Faculty of Pharmacy, Comenius University, Odbojárov 10, 83232 Bratislava, Slovakia; horvath@fpharm.uniba.sk

**Keywords:** benzophenantridine alkaloids, berberine bridge enzyme, (*S*)-*N*-methylcoclaurine-3′-hydroxylase, 3′-hydroxy-*N*-methyl-*(S)*-coclaurine 4′-*O*-methyltransferase, lipoxygenase, Abiotic elicitation, *Eschscholzia californica* Cham., manganese chloride

## Abstract

The basal production of secondary metabolites in medicinal plants is limited. One of the effective approaches that encourages plants to produce a remarkable amount of precious compounds is an application of elicitors. Our work was focused on the elicitation of *Eschscholzia californica* Cham. suspension cultures using various concentrations of MnCl_2_ (5; 10; 15 mg/L) with the aim of evaluating its effect on sanguinarine, chelerythrine, and macarpine production and gene expression of enzymes involved in the biosynthesis of mentioned secondary metabolites (BBE, 4′-OMT, CYP80B1) or in defense processes (LOX). Suspension cultures were exposed to elicitor for 24, 48, and 72 h. The content of alkaloids in phytomass was determined on the basis of their fluorescence properties. The relative mRNA expression of selected genes was analyzed using the ΔΔCt value method. PCR products were evaluated by melting curve analysis to confirm the specific amplification. Our results demonstrated that *Eschscholzia californica* Cham. cell suspension cultures evince sensitivity to the presence of MnCl_2_ in growth media resulting in the increased production of benzophenanthridine alkaloids and gene expression of selected enzymes. Manganese chloride seems to be a potential elicitor supporting natural biosynthetic properties in plant cell cultures and can be applied for the sustained production of valuable secondary metabolites.

## 1. Introduction

The role of secondary metabolites in plants was not clear for a long time. Secondary metabolites are generally not essential for the growth, development, or reproduction of plants but play a major role in the adaptation of plants to their surrounding environment and in overcoming stress [1,2,3]. The involvement of plant secondary metabolites in defense processes is not their single benefit; many of them possess immense biological activities. The effects of medicinal plants on human or animal organisms have been discovered in ancient times based on empirical experience [4] before the isolation and characterization of the active compounds responsible for their therapeutic effect [5,6]. Although current drug development is based on the synthetic preparation of active compounds, the importance of natural products in pharmacy cannot be fully replaced by synthetic drugs. Isolation of desired natural products from wild-growing or cultivated plants is often the only way to obtain appreciable amounts because their chemical synthesis is either extremely difficult or non-profitable [7]. On the other hand, the content of valuable secondary metabolites in medical plants is usually low (less than 1% dry weight), as these substances play no fundamental role in the maintenance of plant life processes [8]. The commercial importance of secondary metabolites has increased in recent years regarding possibilities to obtain them by means of altering the metabolism of the plant cell cultures [9,10]. Enhancement of secondary compounds production in plant cell cultures by elicitation is one of the few recent strategies that has found commercial application [11,12]. The positive effect of abiotic elicitors on the synthesis of phenolic acids (especially rosmarinic acid) and anthocyanins has been investigated in purple basil leaves (*Ocimum basilicum* L.). Elicitors evinced a significant impact not only on levels of bioactive compounds but also on biological activities, especially the anti-inflammatory properties of purple basil leaves [13,14]. In another study, the effect of methyl jasmonate has been evaluated in respect of total phenolic content, isothiocyanate content, and antioxidant activity in radish sprout (*Raphanus sativus* L.) [15]. Elicitation techniques seem to be a good alternative to the genetic modification of plants and provide a way to achieve higher yields of plant secondary metabolites due to controlled stress conditions [16] either in whole plants [13,14,15] or in plant cell tissue cultures [17,18,19].

*Eschscholtzia californica* Cham. (California poppy) is a member of the Papaveraceae family, known for the production of a broad spectrum of benzylisoquinoline alkaloids (BIAs). Among them, the subgroup of quarternary benzo[c]phenanthridins, commonly isolated from plants of the Fumariaceae, Papaveraceae, Ranunculaceae, and Rutaceae families, display promising biological activities. Quaternary ammonium salts of benzophenanthridine alkaloids often possess antimicrobial [20], antiviral [21], and anti-inflammatory activity [22,23], supporting their use in both traditional and modern medicine [24]. Sanguinarine, chelerythrine, macarpine, and chelirubine have been identified as potential anticancer drugs owing to their strong antiproliferative effect based on apoptosis [25,26,27].

The biosynthesis of benzylisoquinoline alkaloids (BIAs) shares a common pathway beginning with the condensation of two molecules of L-tyrosine derivatives resulting in the formation of *(S)*-norcoclaurine. A series of methylation and hydroxylation reactions convert *(S)*-norcoclaurine to *(S)*-reticuline, the crucial intermediate common to the biosynthesis of all BIAs [28]. The broad range of BIAs produced by California poppy predetermine this plant as a model for the study of the gene expression of enzymes participating in different stages of their biosynthesis. Over the past several decades, the research has resulted in complete or near-complete characterization of enzymes and their corresponding genes involved in the biosynthesis of BIAs [29,30,31,32,33].

The present study was carried out to demonstrate the elicitation effect of manganese chloride on the formation of the final products sanguinarine, chelerythrine, and macarpine in suspension cultures of California poppy. Further, the effect of heavy metal elicitation was evaluated on the basis of gene expression of the berberine bridge enzyme (BBE) (EC 1.21.3.3), (*S*)-*N*-methylcoclaurine-3′-hydroxylase (CYP80B1) (EC 1.14.13.71), 3′-hydroxy-*N*-methyl-(*S*)-coclaurine 4′-O-methyltransferase (4′-OMT) (EC 2.1.1.116), and lipoxygenase (LOX) (EC 1.13.11.12).

## 2. Results

### 2.1. Influence of Manganese Chloride Elicitation on Benzophenanthridine Alkaloids Production

**Figure 1:**
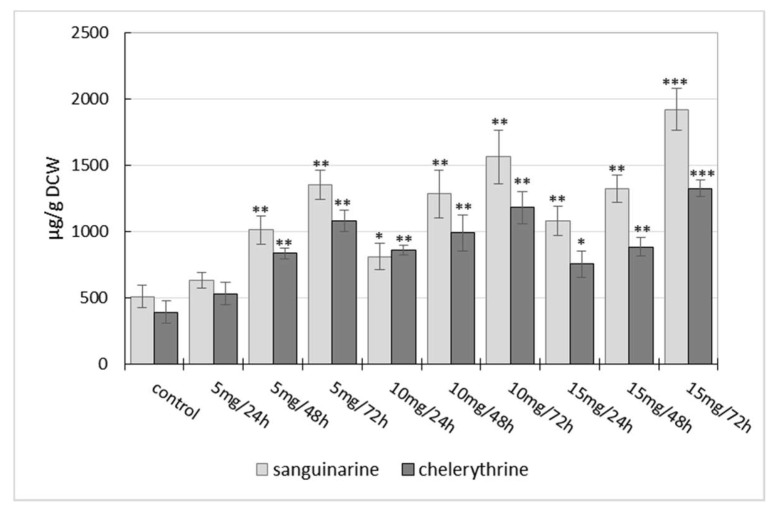
Time- and dose-dependent course of sanguinarine and chelerythrine production in cell suspension cultures of California poppy after MnCl_2_ treatment. Values are means ± SD from triplicate experiments (*n* = 3). * *p* ≤ 0.05, ** *p* ≤ 0.01, *** *p* ≤ 0.001. DCW = dry cell weight.

**Figure 2:**
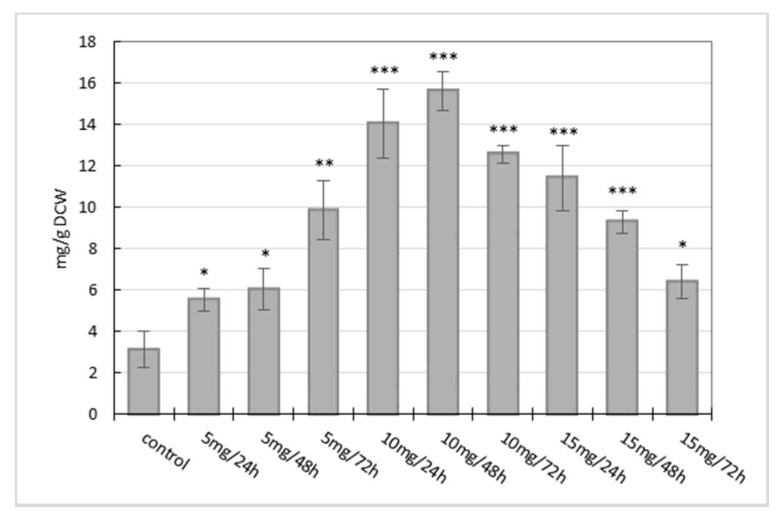
Time- and dose-dependent course of macarpine production in cell suspension cultures of California poppy after MnCl_2_ treatment. Values are means ± SD from triplicate experiments (*n* = 3). * *p* ≤ 0.05, ** *p* ≤ 0.01, *** *p* ≤ 0.001.

The effect of three different concentrations of manganese chloride (5; 10; 15 mg/L) on benzophenanthridine alkaloid production in suspension cultures of California poppy was evaluated. At first, methanolic extracts prepared from cell suspension cultures were subjected to TLC analysis of alkaloids. The TLC results after MnCl_2_ elicitation showed increased fluorescence of spots corresponding to sanguinarine, chelerythrine, and macarpine in comparison with control samples. The fluorometric quantification of each isolated alkaloid confirmed their increased production in plant biomass induced by elicitation. As can be seen in Figure 1, the content of sanguinarine and chelerythrine in suspension cultures increased proportionally to the length of elicitor treatment and its concentration. Sanguinarine and chelerythrine accumulation in cells of suspension cultures peaked at 1924.03 ± 160.30 µg/g dry cell weight (DCW) and 1327.93 ± 61.71 µg/g DCW, respectively, after 72 h of elicitor treatment at a concentration of 15 mg/L of MnCl_2_ (Figure 1). It is approximately a 4- and 3.5-fold increase, respectively, of basal production of both alkaloids in comparison to non-elicited suspension cultures. In the case of macarpine, its production in non-elicited suspension cultures was significantly higher than the production of sanguinarine and chelerythrine. Manganese chloride evinced a strong effect on the formation of macarpine in elicited suspension cultures at all used concentrations. The highest macarpine accumulation was obtained after 24 h (14.04 ± 1.68 mg/g DCW) and 48 h (15.64 ± 0.93 mg/g DCW) of elicitation with MnCl_2_ at a concentration of 10 mg/L. In addition, it was observed that a high concentration of elicitor (15 mg/L) showed a different time-course of macarpine accumulation in cells than lower doses of elicitor. Whereas lower elicitor concentrations (5 and 10 mg/L) induced macarpine production in a time- and dose-dependent course, a high elicitor concentration (15 mg/L) caused a decrease in macarpine content in the case of long-term treatment (Figure 2).

### 2.2. Evaluation of Gene Expression

**Figure 3:**
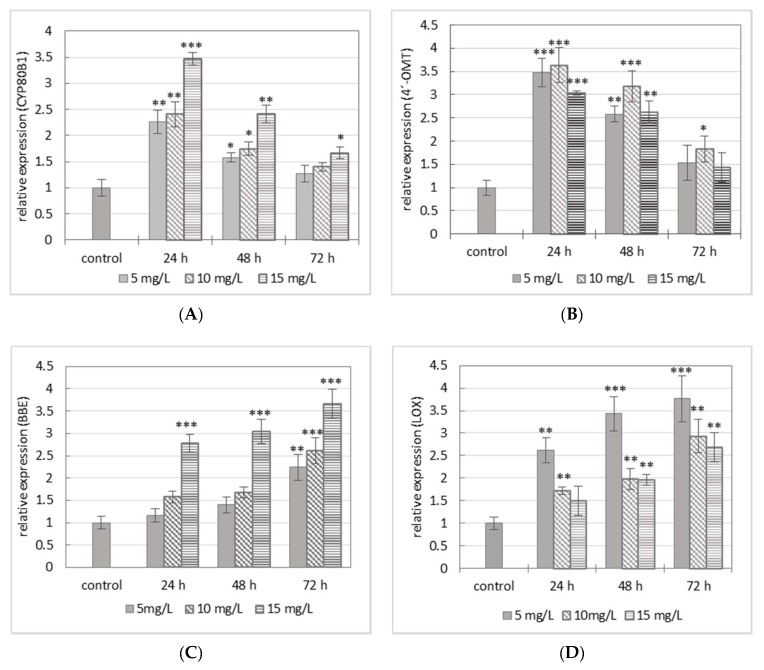
Manganese-chloride-induced expressions of *CYP80B1* (**A**); *4′-OMT* (**B**); *BBE* (**C**); and *LOX* (**D**) genes in cell suspension cultures of California poppy. The relative expression level shows the values standardized by that of the control (non-elicited sample) as 1. Values are means ± SD from triplicate experiments (*n* = 3). * *p* ≤ 0.05, ** *p* ≤ 0.01, *** *p* ≤ 0.001.

The accumulation of sanguinarine, chelerythrine, and macarpine in cell suspension cultures of California poppy requires the activation of enzymes participating in their biosynthetic pathways. In elicited cultures, the expression of four genes (*CYP80B1, 4′-OMT, BBE*, and *LOX*) was assessed. Three of them are related to BIA biosynthesis (CYP80B1, 4′-OMT, and BBE), and lipoxygenase plays a role in the formation of jasmonic acid derivatives, which are signal molecules mediating plant response to stress. In the final evaluation, manganese chloride induced gene expression of studied enzymes in the case of all used concentrations of MnCl_2_. However, the analysis of induction kinetics showed differences in their expression profiles. The relative expression of *CYP80B1* and *4′-OMT* genes reached maximum within 24 h in cells elicited with 15 mg/L (*CYP80B1*) and 10 mg/L (*4′-OMT*) of MnCl_2_ and gradually decreased at longer-lasting elicitation (Figure 3A,B). The gene expressions of the other two studied genes, *BBE* and *LOX*, showed different kinetics than the above-mentioned genes. The increase of the relative gene expressions of *BBE* and *LOX* was slower and prolonged; the values peaked only after 72 h of elicitor treatment. As regards the concentration of elicitor, the gene expression of *BBE* was rather stimulated by a higher dose (15 mg/L), whereas the expression of the *LOX* gene was induced by a lower dose (5 mg/L) of MnCl_2_ (Figure 3C,D).

## 3. Discussion

Cell cultures have been established from many medicinal plants, but they are not often able to produce required secondary metabolites in sufficient amounts. Elicitation is one of several approaches used to enhance the production of secondary metabolites in their natural sources. The pharmaceutical importance of alkaloids produced by species of the Papaveraceae family is undoubted and is not only due to the production of morphinans but also to the production of benzophenanthridine alkaloids. The anti-inflammatory and disinfecting effect of these alkaloids have been widely used in oral hygiene for the prevention or treatment of gingivitis and other inflammatory conditions. However, recent studies have broadened the pharmacological profile of benzophenanthridine alkaloids by antiproliferative [25], pro-apoptotic [27], and neuroprotective [34] effects. Taking into account all of the mentioned biological activities, benzophenanthridine alkaloids can become promising drugs in therapy of tumors and brain injury, although their isolation is still dependent on isolation from intact plant.

The effect of heavy metal elicitation on benzophenanthridine alkaloid production in cell suspension cultures of the Papaveraceae species has been evaluated only in a few articles [35,36,37]. In our experiment, we used three different concentrations of elicitor. Our results show that the optimal dose of MnCl_2_ for alkaloid yield maximization is different for sanguinarine/chelerythrine and for macarpine. The maximum of sanguinarine (4-fold) and chelerythrine (3.5-fold) accumulation was achieved when suspension cultures were exposed to MnCl_2_ at a concentration of 15 mg/L for 72 h. In the case of macarpine, a different time- and dose-dependent course of accumulation was observed. While sanguinarine and chelerythrine production continually increased during elicitation, macarpine accumulation reached maximum after 48 h of elicitation at a concentration of 10 mg/L of MnCl_2_. The application of a high dose of elicitor (15 mg/L) did not induce a further increase of macarpine accumulation and with a prolonged elicitation time its production declined. Manganese chloride has also been evaluated as an elicitor enhancing the production of plumbagin in hairy root cultures of *Plumbago indica* [38] as well as thymol and thymoquinone in suspension cultures of *Nigela sativa* [19]. The effect of AgNO_3_ and CdCl_2_ on sanguinarine production in suspension cultures of California poppy was evaluated in Bilka et al. 2013 [37]. The tested heavy metal salts increased sanguinarine production approximately 5 times in comparison to a non-elicited control sample after 48 h of elicitor treatment. Another study revealed an elicitation effect of various heavy metal salts on the production of gymnemic acid in suspension cultures of *Gymnema sylvestre*, where the optimum time period for elicitation was found to significantly depend on the toxicity of the used heavy metal salt [11].

In the present work, the elevated benzophenanthridine alkaloids production in elicited suspension cultures of California poppy and the gene expression of three enzymes involved in their biosynthesis were evaluated. CYP80B1 and 4′-OMT catalyze the two last steps in the formation of (*S*)-reticuline, which is a central intermediate in the biosynthesis of different types of BIA. BBE converts (*S*)-reticuline to (*S*)-scoulerine as the first step in sanguinarine formation. In our study, the expression level of *CYP80B1* and *4′-OMT* increased during 24 h in the case of all used concentrations of MnCl_2_. After 24 h, the gene expression of both enzymes decreased. In contrast, the expression of *BBE* gene showed an increasing time course throughout elicitation. The gene expression patterns of the tested enzymes are in compliance with their gene expressions studied in various plant tissues of *Eschscholzia californica* seedlings treated with methyl jasmonate (MJ) [30]. Cho et al. 2008 investigated the effect of MJ, salicylic acid, and yeast extract on protein expression in suspension cultures of *Eschscholzia californica* [39]. The results showed differences in the protein expression profiles of the tested enzymes in the case of separate, sequential, and simultaneous treatment. Among the used elicitors, only MJ evinced a similar effect on the protein expression of CYP80B1, 4′-OMT, and BBE as MnCl_2_ on their gene expression.

Lipoxygenase is related to the formation of secondary mediators responsible for the activation of defense processes triggered by different stress factors (biotic and abiotic) [17,18,40]. Abiotic elicitation of California poppy suspension cultures with MnCl_2_ resulted in elevation of *LOX* gene expression in a time-dependent manner. The highest relative expression of *LOX* gene was detected after 72 h of elicitation and the most efficient concentration was decided to be 5 mg/L of MnCl_2_. Although higher doses of elicitor (10 and 15 mg/L) also elevated the relative expression of *LOX* gene, it did not reach the same level as at 5 mg/L. LOX activity was successfully stimulated in opium poppy cell suspension cultures elicited with MJ (100 µmol/L) and fungal elicitor from *Botrytis cinerea*. Both elicitors resulted in the highest induction of LOX activity in the 10th h of elicitation [17]. On the other hand, elicitation of California poppy suspension culture with the same elicitors evinced a different course of change in specific LOX activity than in opium poppy. The highest induction of specific LOX activity was achieved at 72 h of elicitor treatment [18]. The time course analysis of *LOX* gene expression determined in our study showed the same kinetic profile as that of alterations of specific LOX activity in the previously mentioned study. Based on these results, we can suppose that LOX plays an important role in the activation of defense processes triggered by abiotic elicitors resulting in the formation of benzophenanthridine alkaloids.

## 4. Materials and Methods

### 4.1. Preparation of Suspension Cultures and Elicitation with MnCl_2_·4H_2_O

California poppy suspension cultures were prepared from friable callus proliferated on a paper bridge in Murashige–Skoog (MS) medium supplemented by kinetin (2 mg/L) and 1-naphtylacetic acid (NAA) (1 mg/L). Callus cultures were transferred into Erlenmeyer flasks with 50 mL of sterile liquid MS medium and maintained on an orbital shaker (rpm 110) for one month under 16/8 h white light/dark period (22,000 lux) at 24 °C and a relative humidity of 70%. After one month of subcultivation, the cell biomass was transferred into Erlenmeyer flasks with 50 mL of fresh MS growth medium and cultivated for the next 14 days under the same conditions mentioned above.

### 4.2. Elicitor Preparation

Manganese chloride tetrahydrate (MnCl_2_·4H_2_O) was used for abiotic elicitation of California poppy suspension cultures. Metal salt was prepared as a stock in distilled water at a concentration of 0.15 g/L. The solution was added to suspension cultures aseptically using 0.22 µm syringe membrane filters (Millipore, Merck, Germany) at final concentrations of 5, 10, and 15 mg/L in growth media [19]. All elicited samples were prepared in triplicate. Plant material was harvested after 24, 48, and 72 h of elicitor treatment. Cells were finally separated from growth medium by vacuum filtration, lyophilized, and stored at −20 °C.

### 4.3. Alkaloids Determination and Quantification

Lyophilized ground suspension cultures (0.1 g) were suspended in 10 ml of methanol and extracted overnight on an orbital shaker (110 rpm). Acquired extracts were subsequently centrifuged at 10,000× *g* for 15 min to separate the supernatant from cell debris. Supernatants of each sample containing benzophenanthridine alkaloids were subjected to TLC and spectrofluorometric analysis. Methanolic extracts of elicited suspension cultures (100 µL) were separated on TLC plates (Silicagel 60, Merck, Darmstadt, Germany) using mobile phase: methanol:chloroform:ammonia 60:35:5 (*v*/*v*/*v*). Detection of developed chromatogram plates was performed by transilluminator Camag Reprostar II (Muttenz, Switzerland) equipment. The benzophenanthridine alkaloids sanguinarine and chelerythrine were identified on the basis of comparison with reference standards obtained from Sigma-Aldrich (St. Louis, MO, USA). Macarpine was isolated from methanolic extract of California poppy cell suspension cultures and purified by column chromatography (30 × 1.5 cm) on Silicagel 60Å (Merck, Germany) using mobile phase chloroform:methanol:benzene 70:15:15 (*v*/*v*/*v*). The elution of benzophenanthridine alkaloids was controlled by a UV lamp. Fractions containing macarpine were collected and mobile phase was evaporated to dryness. The identity and purity of isolated macarpine was verified by ^1^H-NMR (Varian NMR System 600, Santa Clara, CA, USA, data are not shown) and compared with literature data [41]. Purified macarpine was used as a reference standard.

Spots corresponding to standards were separately removed from TLC plate and suspended in 1 mL of 50% UV ethanol containing 0.02 mol/L NaOH, extracted for 1 h, and centrifuged at 6000 rpm for 15 min. Volumes of obtained supernatants were adjusted to 10 mL by 50% UV ethanol containing 0.02 mol/L NaOH.

Isolated benzophenanthridine alkaloids were finally quantified using a fluorescent-based assay at maximum excitation/emission wavelengths (Luminescence spectrometer LS-30, Perkin-Elmer, Beaconsfield, UK) λ_ex_/λ_em_ = 324/408 nm for sanguinarine [18], λ_ex_/λ_em_= 294/407 nm for chelerythrine [42], and λ_ex_/λ_em_ = 269/420 nm for macarpine (determined by a pre-scan assay of the luminescence spectrometer). Fluorescence intensity was converted to alkaloid concentration by the obtained reference standards’ calibration curves. The final content of alkaloids was expressed in µg or mg per gram of dry cell weight (DCW).

### 4.4. Total RNA Isolation and Quantitative RT-PCR

Total RNA was isolated from the 100 mg of cell culture using RNAzol RT (Molecular Research Center INC., Cincinnati, USA) and converted into complementary DNA (cDNA) using the PrimeScript RT Reagent Kit (Takara, Kusatsu, Japan) following the protocols of the manufacturers. Amplification and detection of cDNA of reference and target genes were performed on a 7300 Real-Time PCR System (Applied Biosystems, Singapore) using HOT FIREPol EvaGreen qPCR Mix Plus (ROX) (Solis BioDyne, Tartu, Estonia). The relative mRNA expressions of *BBE*, *CYP80B1, 4′-OMT*, and *LOX* were analyzed using the ΔΔCt value method [43]. PCR products were evaluated by melting curve analysis to confirm the specific amplification. β-actin was used as a reference gene. The sequences of used primers are listed in Table 1 and come from articles of Ikezawa et al. 2007 [30] and Mishra et al. 2013 [44].

### 4.5. Statistical Analysis

All of the experiments were performed in triplicate and data were expressed as means ± standard deviations. The paired sample Student’s *t*-test was used to determine significant (*p* ≤ 0.05) differences.

## 5. Conclusions

The present study was focused on the evaluation of the effect of manganese ions on benzophenanthridine alkaloid formation in suspension cultures of California poppy and the study of its connection to the gene expression of enzymes involved in alkaloid biosynthesis and defense processes evoked by abiotic stress. The results have shown that the presence of MnCl_2_ in the growth media of in vitro cultures supports natural biosynthetic properties of cells, resulting in an increase of benzophenanthridine alkaloid production in a time- and dose-dependent manner. In connection to alkaloid accumulation, an increased gene expression of *CYP80B1, 4′-OMT*, and *BBE* was confirmed. The elevated gene expression of *LOX* suggests its involvement in the activation of defense processes triggered by heavy metals in plant cells. It can be concluded that manganese chloride seems to be an auspicious elicitor that stimulates natural biosynthetic properties of California poppy cell suspension cultures leading to significant benzophenanthridine alkaloids production.

## Figures and Tables

**Table 1:** Nucleotide sequences of primers.

Primer Name	Oligonucleotide Sequences (5′- to 3′-)
CYP80B1	forward TCAAACAGTGGTAGGCGAGAGA reverse CAATGGAGTTGGTGGGTGAA
BBE	forward GAGATTAGTAGGAGTTGGGGTGAGA reverse ATTGGAGGGATACTTTGTGGATG
4′-OMT	forward CCTAGAAGAGGAATCAGAACATCCA reverse TCACTTCTCTCCCTTCCACCA
LOX	forward ATTGGGAAAATGACGATGGAAAA reverse CTACGTTATCCCTTGTAAACCATTC
β-actin	forward GGTATTGTGCTGGATTCTGGTG reverse GTAGGATTGCGTGGGGTAGTG
